# The intracellular domain of UNC5B facilities proliferation and metastasis of bladder cancer cells

**DOI:** 10.1111/jcmm.16172

**Published:** 2020-12-20

**Authors:** Yexiang Huang, Zhe Zhang, Miao Miao, Chuize Kong

**Affiliations:** ^1^ Department of Urology The First Hospital of China Medical University Shenyang China

**Keywords:** apoptosis, bladder cancer, mass spectrometry, metastasis, UNC5B

## Abstract

The intracellular domain of UNC5B contains both death domain and caspase‐3 cleavage site, and is regarded as a functional domain that mediates apoptosis. However, in our previous studies, we found that the death domain of UNC5B in bladder cancer cells could not be activated to promote apoptosis. In this study, different UNC5B truncates (residue 399‐945, residue 412‐945) were created to explore whether the caspase‐3 cleavage site (site 412), as another potential functional domain of its intracellular portion, could be activated to induce apoptosis in bladder cancer cells. Using mass spectrometry, we acquired a comprehensive and detailed identification of differentially expressed proteins by overexpressing UNC5B and its truncates. Protein‐protein‐interaction (PPI) network analysis was also applied to investigate the aggregation of related proteins and predict the functional changes. EDU assay, apoptosis, xenograft tumour implantation, migration, invasion and tumour metastasis were performed to comprehensively identify the effects of UNC5B truncates on bladder cancer cells. We demonstrate that the intracellular domain of UNC5B promotes cell proliferation in vitro and tumour formation in vivo, by binding to a large number of ribosomal proteins. The overexpression of intracellular domain also facilitates cells to migrate, invade and metastasize by interacting with fibronectin, beta‐catenin and vimentin. In addition, we reveal that overexpressing the intracellular domain of UNC5B cannot bind or activate cleaved caspase‐3 to trigger apoptosis in bladder cancer cells.

## 1 INTRODUCTION

Bladder cancer (BC, bladder urothelial carcinoma) is a very common type of urinary system cancer in developed countries.^1^, ^2^, ^3^, ^4^ Despite of the development of surgical technology, the incidence and mortality of BC have not decreased in the last decade.^5^, ^6^, ^7^ In view of the diversity of biological behaviour and molecular phenotypes,^8^, ^9^, ^10^ BC is characterized by multiple germinal centres, easy recurrence and metastasis. In addition, muscle‐invasive bladder cancer (MIBC) is not sensitive to traditional radiotherapy^11^, ^12^ or chemotherapy.^13^ Biological therapy that activates tumour suppressor genes has proven to be effective in some recent studies.^14^, ^15^, ^16^, ^17^

The transmembrane protein uncoordinated‐5 homolog B (UNC5B) is verified to inhibit cell proliferation in some types of tumours.^18^, ^19^, ^20^, ^21^ The intracellular domain of UNC5B contains both caspase‐3 cleavage site and death domain (residue 865‐943).^22^, ^23^ As reported, the death domain interacts with death‐associated protein kinase (DAPK) to mediate apoptosis,^24^, ^25^, ^26^, ^27^ and the caspase‐3 cleavage site is also a potential activation target of p53‐mediated apoptosis.^28^, ^29^ However, in our previous studies, the cotransfection of UNC5B and DAPK in BC cells did not cooperate to promote apoptosis, but significantly reversed the anti‐tumour effect of DAPK.

In this study, UNC5B truncates (residue 399‐945, residue 412‐945) were created and transfected to explore whether the caspase‐3 cleavage site of UNC5B, as another potential functional domain of its intracellular portion, could be activated to induce apoptosis in BC cells. Using mass spectrometry, we acquired a comprehensive and detailed identification of differentially expressed proteins by overexpressing UNC5B and its truncates. Protein‐protein‐interaction (PPI) network analysis was also applied to investigate the aggregation of related proteins and predict the functional changes. EDU assay, apoptosis, xenograft tumour implantation, migration, invasion and tumour metastasis were performed to comprehensively identify the effects of UNC5B truncates on BC cells.

We demonstrate that the intracellular domain of UNC5B promotes cell proliferation and tumour formation, by binding to a large number of ribosomal proteins. The overexpression of intracellular domain also facilitates BC cells to migrate, invade and metastasize by interacting with fibronectin, beta‐catenin and vimentin. In addition, we reveal that overexpressing the intracellular domain of UNC5B cannot bind or activate cleaved caspase‐3 to trigger apoptosis in bladder cancer cells.

## 2 METHODS AND MATERIALS

### 2.1 Plasmids, virus productions and reagents

UNC5B(399‐945aa)‐3Flag overexpression lentivirus and UNC5B(412‐945aa)‐3Flag overexpression lentivirus were synthesized by Obio Technology Co. Ltd. Tables 1 and 2 indicated the composition reports. Full‐length UNC5B‐Flag overexpression lentivirus was purchased from GenePharm Co. Ltd. The Lipofectamine 3000 Reagent (Life Technologies Co. Ltd.) was used for these plasmids and related transfection.

**TABLE 1:**
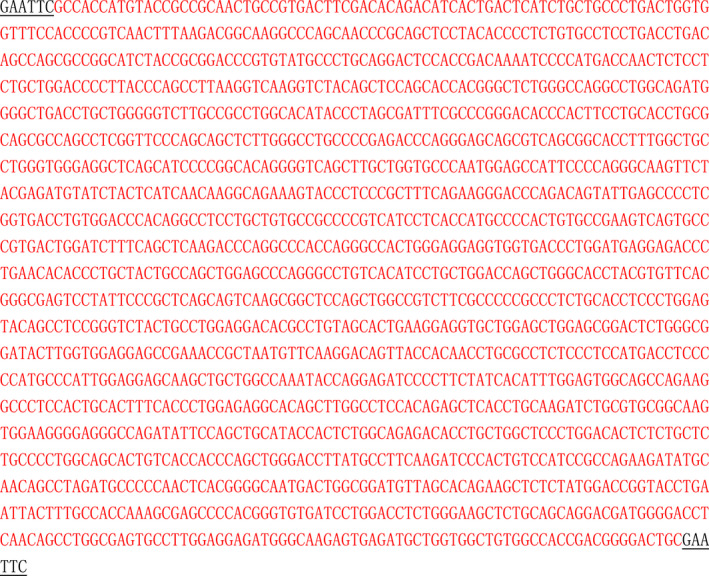
399‐945'UNC5B sequence analysis

**TABLE 2:**
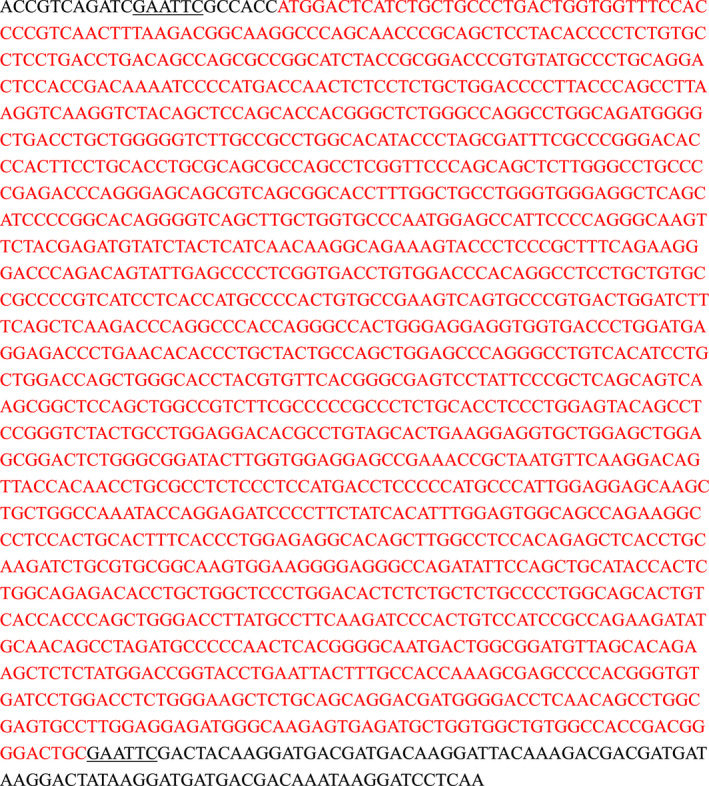
412‐945'UNC5B sequence analysis

Antibodies against UNC5B (ab104871, ab54430), caspase‐3 (ab179517), mutant P53 (ab32049) and BCL‐2 (ab32124) were purchased from Abcam. Antibodies against cleaved PARP (D64E10), S6 Ribosomal Protein (5G10), UNC5B (D9M7Z) and beta‐actin (3700S) were obtained from Cell Signaling Technology (CST). Antibodies against Fibronectin, beta‐catenin, vimentin, N‐CA and E‐CA were also obtained from the Epithelial‐Mesenchymal Transition (EMT) Antibody Sampler Kit (9782T, CST). Antibody against FLAG (F1804) was obtained from Sigma‐Aldrich Co. Ltd. Antibody against P53 (21891‐1) was purchased from Proteintech Co. Ltd. UNC5B (ab54430, Abcam) was used for immunofluorescence and flow cytometry. UNC5B (D9M7Z) was used as a mixture reagent in Co‐IP. Universal Magnetic Co‐IP Kit (54002, Active Motif) was purchased from Active Motif Co. Ltd. Fast silver staining kit (P0017S, Beyotime) was purchased from Beyotime Co. Ltd.

### 2.2 Western blot analysis

Cells were washed three times with ice‐cold PBS, homogenized at 4°C in 10 volumes (w/v) of RIPA lysis buffer (Beyotime), and 1 mM PMSF mixture for 30 minutes. The lysates were gathered after centrifuging for 30 minutes (13 000 rpm) at 4°C. After the normalization and denaturation of each sample, 50 µg of total protein was loaded and separated with 12% SDS‐PAGE, and then transferred onto PVDF membrane (Millipore). After blocking with 5% skimmed milk powder in TBST (TBS containing 0.1% Tween‐20), the membranes were incubated with primary antibody (1:1000) overnight at 4°C. After being washed, the membranes were incubated in secondary antibody (1:3000‐1:5000) for two hours at 37°C. The membranes were then washed three times. The immunoreactive bands were examined by a Microchemi 4.2 device (Bio‐Rad) using an enhanced chemiluminescent (ECL) reagent kit.

### 2.3 Co‐immunoprecipitation

In general, we mixed a complete whole‐cell lysis buffer of protease inhibitor cocktail, deacetylase inhibitor, phosphatase inhibitors, PMSF and cell lysis buffer to extract protein from each cell sample. Centrifuge the mixture of cell suspension for at least 10 minutes at 4°C (1500 rpm). We mixed protease inhibitor cocktail, deacetylase inhibitor, phosphatase inhibitors, PMSF and cell lysis buffer to make a complete whole‐cell lysis buffer,and used this lysis buffer to extract protein from each cell sample. We then added the lysis buffer to cell samples and centrifuged the mixture of cell suspension for 10 minutes at 4°C (1500 rpm). 500 µg whole‐cell extract and 5ug antibody was subsequently combined into a pre‐chilled tube. The tube was then incubated at 4°C on a rotator overnight. 25 µL Protein G magnetic beads were then added to each tube. The mixture of Protein G beads, whole‐cell extract and antibody was incubated for one hour at 4°C on a rotator. We centrifuged the tube at 1500 Crpm for one minute and discarded the supernatant. We then washed beads 4 times with the cell lysis buffer. Finally, we resuspended bead pellet in 20 µl of 2X Reducing Loading Buffer. WB, fast silver staining and mass spectrometry were then applied for further investigation.

### 2.4 Fast silver staining

Fast silver staining kit (P0017S, Beyotime) was used for protein detection of electrophoresis gel. Whole protocol was shown briefly as follows: fixing (20 minutes), 30% ethanol washing (10 minutes), water washing (10 minutes), silver dye sensitizing (2 minutes), water washing (1 minute × 2), silver staining (10 minutes), water washing (1 minute), colour rendering (3‐10 minutes, depending on the gel staining), termination (10 minutes) and washing (5 minutes). Gels were directly sent to Shanghai Applied Protein Technology Co. Ltd. (APT) for further investigation.

### 2.5 Mass spectrometry in proteomics and bio‐informatics analysis

Shanghai Applied Protein Technology Co. Ltd. provided bio‐informatics analysis on mass spectrometry in proteomics. The differently expressed proteins between groups were identified by a label‐free proteomics method. GO analysis, map analysis of KEGG signalling pathway and PPI (protein‐protein‐interaction) network analysis were all included to predict the molecular process and signalling pathways. Cytoscape software (version 3.6.1) was applied to analyse and predict protein binding and interaction.

### 2.6 Cell culture and transfection

BC cell lines UMUC3, 5637 and T24; prostate cancer cell 22RV‐1; renal cancer cell 786‐0 were purchased from the Chinese Academy of Sciences Cell Bank (CASCB) and were cultured in RPMI 1640 medium (HyClone). All cells were incubated in an incubator with 5% CO_2_. We transfected UNC5B and UNC5B‐truncates to cells by lentivirus for at least 60 hours. The stably transfected cells were established by using puromycin for at least 3 weeks. The successful transfection was verified by PCR, WB, flow cytometry and immunofluorescence.

### 2.7 Quantitative real‐time polymerase chain reaction

Trizol reagent (Invitrogen) was used to extract total RNA from cells. By using PrimeScript™ RT Master Mix (Takara Biotechnology), RNA was reverse transcribed into first‐strand cDNA. We then performed real‐time qPCR to detect the mRNA expression of UNC5B and beta‐actin genes using SYBR® Premix Ex Taq™ (Tli RNaseH Plus; Takara Biotechnology Co. Ltd.) and a Thermal Cycler Dice™ Real Time TP800 system (Takara). Table 3 indicated the related primer sequences.

**TABLE 3:** Primer sequences of UNC5B and Actin

Primer name	Primer sequences
UNC5B forward	5′ GTCGGACACTGCCAACTATAC 3′
UNC5B reverse	5′ CCGCCATTCACGTAGACGAT 3′
Actin forward	5′ CATGTACGTTGCTATCCAGGC 3′
Actin reverse	5′ CTCCTTAATGTCACGCACGAT 3′

### 2.8 EDU assay

BeyoClick™EdU‐488 cell proliferation detection kit (C0071S, Beyotime) was used for EDU assay. In brief, the serum‐free 1640 medium was used to prepare 2X EDU working solution. The preheated working solution was added to cells and incubated for 2 hours. Then, we added 4% paraformaldehyde and fixed at room temperature for 15 minutes. The cells were subsequently incubated with 0.3%TritonX‐100 's PBS for 15 minutes. We dissolved one tube of Click Additive in 1.3 mL deionized water to prepare Click Additive Solution. 4.3 mL Click Reaction Buffer, 200 μL CuSO4, 10 μL Azide488 and 500 μL Click Additive Solution were mixed into 5 mL Click reaction solution. Each sample was incubated with 0.5 mL reaction solution to avoid light for 30 minutes. After full washing, the percentage of cells in proliferative phase was detected by flow cytometry.

### 2.9 Cell apoptosis and fluorescence detection by flow cytometry

5 × 10^4^ cells were seeded and cultured, and then were harvested and transferred into 0.5 mL PBS. We subsequently used fluorescein isothiocyanate (FITC) Annexin V Apoptosis Detection Kit (BD Pharmingen) to examine apoptosis. Incubated with propidium iodide (PI) and FITC for 10 minutes, cells were performed on flow cytometry for apoptosis tests.

Similarly, we incubated cells with 4 μg/mL UNC5B in the shading for 30 minutes and then transferred into secondary antibody. Fluorescence detection of FL4‐H signal was applied subsequently. Using FlowJo version 10.0.7 (FlowJo) software, we analysed the data of fluorescence.

### 2.10 Immunofluorescence

5 × 10^4^ cells were seeded and cultured for 48 hours, and washed with PBS. 300 μL 4% paraformaldehyde was used to fix cells. Subsequently, 300 μL triton X‐100 was applied to increase the permeability of the cell membrane. 300 μL BSA buffer was added for blocking for 30 minutes. After the BSA was removed, the cells were incubated at 4°C with primary antibody (1:100 diluted) overnight. Fluorescent labelled second antibody was added to cells at room temperature and incubated with shading for 1 hour, and then, DAPI was added to stain the nucleus. Fluorescence microscope was used to observe and record the images.

### 2.11 Xenograft tumour model

This study was conducted according to the Medical Laboratory Animal Welfare and Ethics Committee of China Medical University. We purchased 10 female BALB/c nude mice of 4‐6 weeks old from Beijing Vital River Experimental Animal Technology Co. Ltd and randomly assigned mice into two groups. We injected 412’5637 and 399’5637 cells separately into the right and left armpits of mice in the first group, NC‐5637 and UN‐5637 cells into the second group. We killed the mice 56 days after the subcutaneous injection. We used the equation (volume = length×width^2^/2) to estimate the tumour size.

### 2.12 Cell migration and invasion

#### Cell migration

We added 500 µL 1640 medium with 10% serum to a 24‐well plate and put the transwell chamber on it, then transferred 200 µL serum‐free culture medium mixed with 3000 cells (5637 or T24) to the chamber. After placing the 24‐well plate in cell incubator for 24 hours, we took it out, washed and stained the chamber with crystal violet staining solution. Using a light microscope, we observed the number of cells passing through the transwell chamber and recorded it.

#### Cell invasion

We inverted the transwell chamber, spread 40 µL of preheated Matrigel evenly on the surface of the outer chamber and then placed the chamber in a 24‐well plate. The above experimental operations were carried out in a cell super purification table. After incubated for 4 hours, the following experiment was applied as described in cell migration.

### 2.13 Tail vein injection

Prepare a 50 mL centrifugal tube and hollow out the centre of the tube cap to form a round hole with a diameter of 0.6 cm. Fill the centrifuge tube with cotton, place the mice in it and straighten the tail. Wipe the tail with alcohol to dilate the tail vein, fix the mouse tail with the left hand and inject the PBS mixed with different cells into the vein. The total amount of injected cells is 2 × 10^6^, and the bubbles in the mixture are emptied before injection. The injection dose should not be more than 1.2 mL or <0.8 mL, lest the mice die of heart failure or pulmonary infarction. Following the above process, we injected 16 BALB/c nude mice and killed the animals 8 weeks after the injection.

## 3 RESULTS

### 3.1 The transfection of the full‐length UNC5B and its truncates to BC cells

**FIGURE 1:**
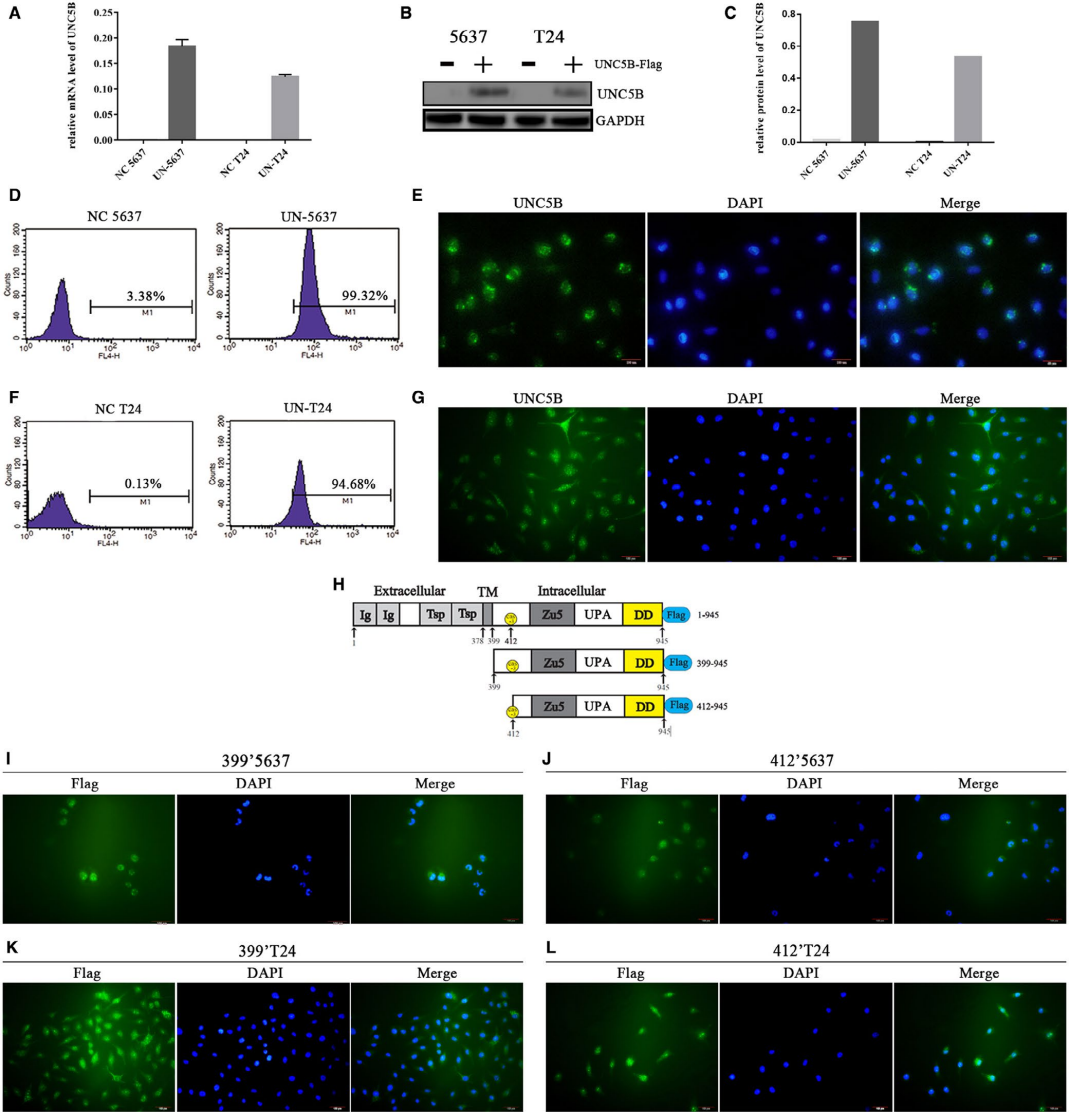
The transfection of the full‐length UNC5B and its truncates to BC cells. A, We transfected Flag‐UNC5B lentivirus and empty vectors to 5637 and T24 cells (abbreviated as UN‐5637/UN‐T24 and NC‐5637/NC‐T24 cells). Relative mRNA expression of UNC5B was analysed in the above cells. B, C, Western blot (WB) analysis of the UNC5B in UN‐5637 and UN‐T24 cells was used to verify the transfection. D, F, We used flow cytometry to verify the transfection of UNC5B in 5637 and T24 cells, respectively. E, G, Immunofluorescence verified the successful transfection of UNC5B in cells. H, Schematic diagram of full‐length UNC5B (top), full‐length intracellular domain of UNC5B (middle: residues 399‐945) and partial intracellular domain of UNC5B (below: residues 412‐945). The caspase‐3 site and the death domain were remarked yellow for their potential function of apoptosis. Blue rounded rectangles were used to label the flag‐tags of lentivirus. I‐L, Immunofluorescence verified the successful transfection of UNC5B truncates (residues 399‐945, residues 412‐945) in the above cells (labelled as 399′5637/412′5637 and 399′T24/412′T24 cells), respectively

In our previous study on BC cells,^20^, ^30^ we found that 5637 cells expressed the highest mRNA level of UNC5B, while T24 cells expressed the lowest. Here, we similarly transfected full‐length UNC5B to 5637 (labelled as UN‐5637) and T24 (labelled as UN‐T24) cells. PCR (Figure 1A), WB (Figure 1B,C), flow cytometry (Figure 1D,F) and immunofluorescence (Figure 1E,G) verified the successful transfection of UNC5B. UNC5B‐truncates overexpression cells (abbreviated as 399’5637/412’5637 and 399’T24/412’T24 cells) were also established by flag‐label lentivirus (Figure 1H). Immunofluorescence (Figure 1I‐L) and sequence analysis (Table 1 and 2) verified the successful induction of the intracellular domains of UNC5B in both cells.

### 3.2 Full‐length UNC5B mildly inhibited cell proliferation and mediated apoptosis, while UNC5B truncates promoted cell proliferation

**FIGURE 2:**
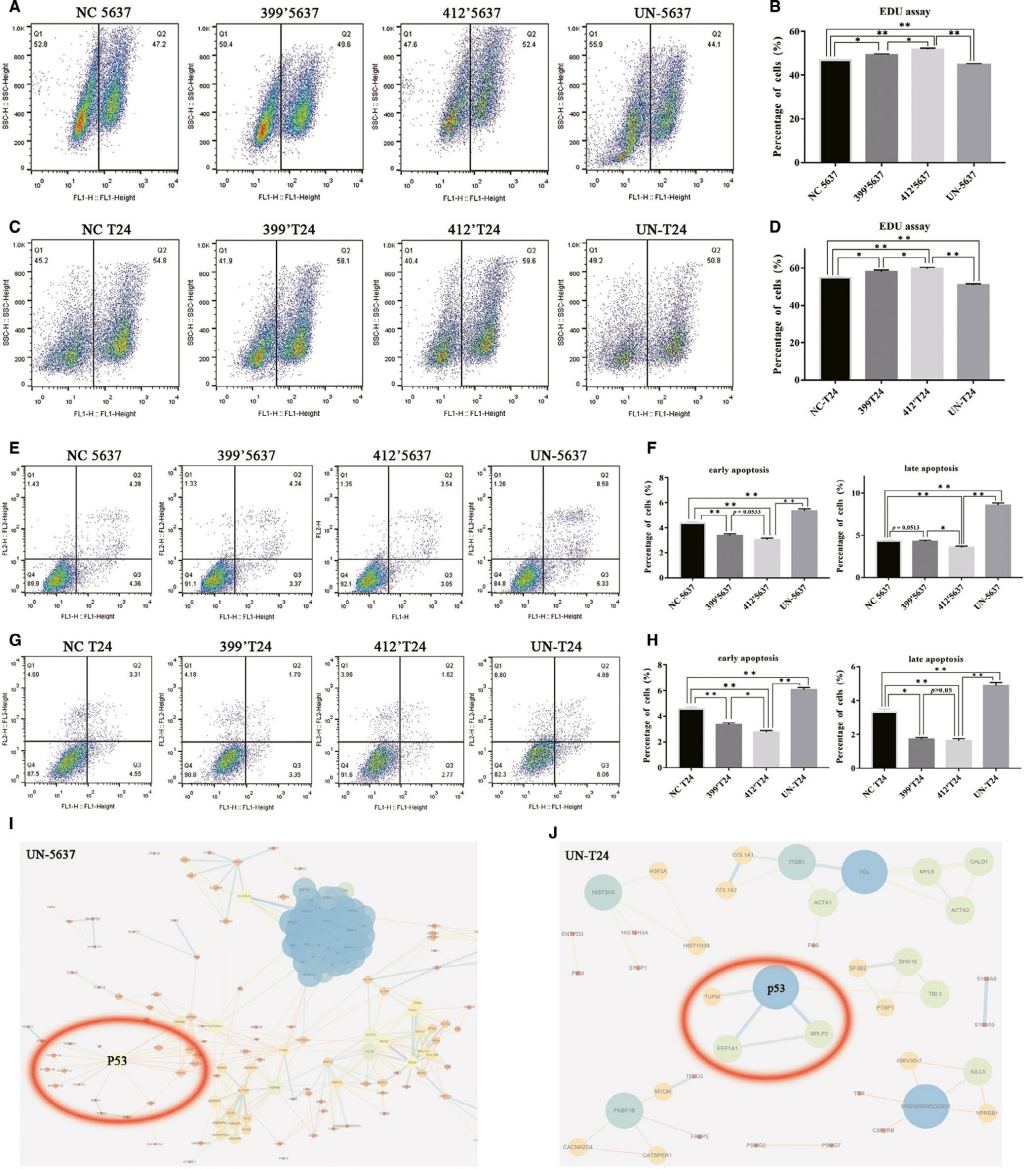
Full‐length UNC5B mildly inhibited cell proliferation and mediated apoptosis, while UNC5B truncates promoted cell proliferation. A, C, EDU assay by flow cytometry indicated the effect of full‐length UNC5B and UNC5B truncates on proliferation in 5637 and T24cells, respectively. B, D, Column statistical chart of the percentage on proliferation phase in 5637 and T24 groups of cells. Student's *t* test. *P < .05. **P < .01 (mean ± SD, n = 3). E, G, Apoptosis tests by flow cytometry indicated the effect of full‐length UNC5B and UNC5B truncates on apoptosis in 5637 and T24cells, respectively. F, H, Column statistical chart of the proportion on early and late apoptotic cells among cells in 5637 and T24 groups. Student's *t* test. *P < .05. **P < .01 (mean ± SD, n = 3). I, J, PPI analysis was shown by overexpressing full‐length UNC5B in 5637 and T24 cells, respectively. The red circle represented the binding of overexpressed UNC5B to P53 and regulatory proteins

Through EDU assay (Figure 2A,C), we observed that a greater proportion of cells were examined at the stage of proliferation by the overexpression of UNC5B truncates both in 5637 and T24 cells. The percentage of cells in the proliferative phase in 412′5637 and 412′T24 cells was 5% higher than that of the NC cells. Comparatively, the percentage of cells in the proliferative phase in the 399′5637 and 399′T24 cells was 2.4% and 3.3% higher than that of the NC cells, respectively. By contrast, the overexpression of full‐length UNC5B decreased the proliferation of both cells. Compared to NC cells, the proportion of the UN‐5637 cells in proliferative phase decreased by 3.1%, and the proportion of the UN‐T24 cells in proliferative phase decreased by 4%. The apoptosis test indicated that UNC5B mediated mild apoptosis by increasing the proportion of both early and late apoptotic cells (Figure 2E,G). However, no significant difference was found in the proportion of apoptotic cells among different groups (The biggest difference was 4.3%, representing the difference of percentage of early apoptotic cells between NC‐5637 and UN‐5637 cells). On the other hand, the pro‐apoptotic effect of full‐length UNC5B on BC cells no longer existed by overexpression of UNC5B truncates in both cells (Figure 2E‐H).

Through fast silver staining (Figure S1A,B) and mass spectrometry, a variety of differentially expressed proteins were identified and aggregated into protein complexes in UN‐5637 and UN‐T24 cells (Figure 2I,J). Multiple pathways took part in the process, including cell cycle regulation, protein transport, programmed cell death and regulation of apoptosis. In our previous study,^30^ we found that UNC5B promoted G2/M phase arrest and inhibited cell proliferation by combining with P53 and CDC14A. Here, we used PPI analysis (Figure 2I,J) to clarify the combination of UNC5B and cell cycle regulatory proteins (such as P53) in UN‐5637 and UN‐T24 cells.

### 3.3 The binding of ribosomal protein to UNC5B truncates was closely related to cell proliferation and tumour formation in mice

**FIGURE 3:**
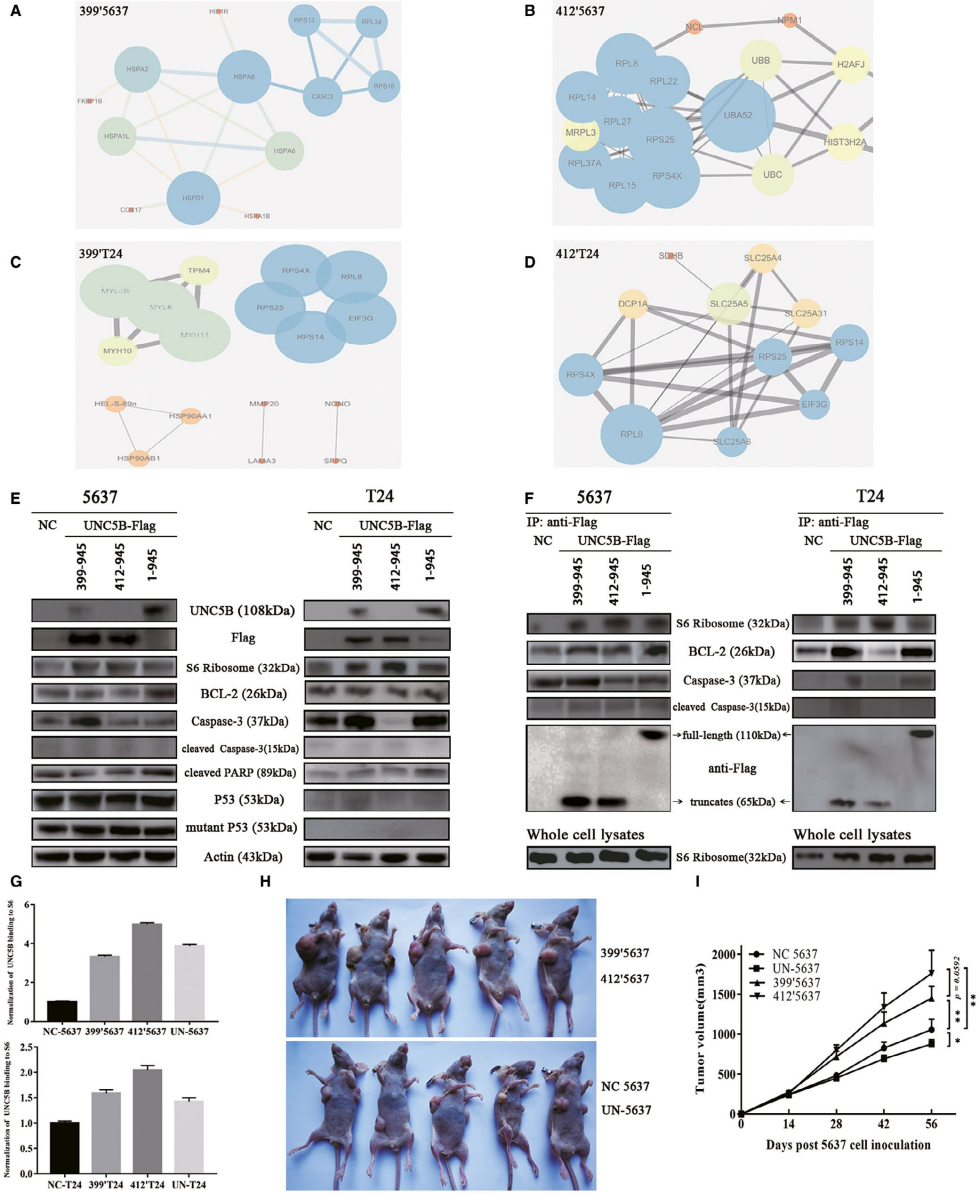
The binding of ribosomal protein to UNC5B truncates was closely related to cell proliferation and tumour formation in mice. A–D, PPI analysis was shown by overexpressing different intracellular domains of UNC5B (A, C: residues 399‐945, B, D: residues 412‐945) in 5637 and T24 cells, respectively. Ribosomal proteins aggregated into complexes were marked dark blue. E, WB analysis demonstrated that S6 ribosomal protein was increased by transfecting UNC5B truncates and full‐length UNC5B. The expression of cleaved PARP was elevated in UN‐5637 and UN‐T24 cells. However, no band corresponding to cleaved caspase‐3 was found in both groups of cells. Moreover, there was no change in the expression of P53 or mutant P53. F, S6 ribosome protein and BCL‐2 were co‐immunoprecipitated by CO‐IP tests. Cleaved caspase‐3 was seldomly co‐immunoprecipitated in 5637 group of cells, but not in T24 group. Co‐IP tests also revealed that the overexpression of UNC5B and UNC5B truncates increased the binding of S6 ribosomal protein. More binding of BCL‐2 was also found in 399′5637, UN‐5637, 399′T24 and UN‐T24 cells, but not in 412′T24 cells. G, The normalization of the binding of UNC5B to S6 ribosome protein was shown in CO‐IP tests (top: 5637 cells; below: T24 cells). H, 399′5637 and 412′5637 cells, UN‐5637 and NC‐5637 cells were separately injected into the bilateral armpits of mice of two groups. The formation of tumours was observed 56 days after the injection. I, Image of tumour volume was shown after the injection of the different cells. The formula (volume = length*width^2^/2)was applied to estimate the tumour size. Student's *t* tests. *P < .05. **P < .01 (mean ± SD, n = 5)

Differentially expressed proteins between groups were also analysed in cells overexpressing UNC5B truncates. Unlike the PPI analysis in UN‐5637 and UN‐T24 cells (Figure 2I,J), the interaction of UNC5B with P53 (and some other regulatory proteins) no longer existed in 399′5637, 412′5637, 399′T24 or 412′T24 cells (Figure S1C‐F). On the other hand, a large number of homogeneous ribosomal proteins (marked dark blue) aggregated into complex and potentially promoted cell proliferation in the above cells (Figure 3A‐D). In detail, more ribosomal proteins were identified in 412’5637 cells than that in 399’5637 cells (Figure 3B vs Figure 3A). This trend was also seen between 412’T24 and 399’T24 cells (Figure 3D vs Figure 3C). Additionally, the number of identified ribosomal proteins was consistent with the result of EDU assay (Figure 2A‐D) by overexpressing UNC5B truncates. Except for the ribosomal protein and histone that bound to UNC5B truncates, few other proteins were co‐immunoprecipitated or formed protein complex to affect cell proliferation.

As both caspase‐3 site and death domain were located in the UNC5B truncates, we examined the protein expression of total caspase‐3, cleaved caspase‐3, cleaved PARP, BCL‐2, p53 and mutant P53 to clarify the potential activation of caspase‐3 and apoptosis. Unexpected, no band corresponding to cleaved caspase‐3 was found through WB in both groups. However, the expression of cleaved PARP was increased in UN‐5637 and UN‐T24 cells, which was consistent with the result of apoptosis (Figure 2E ~ 2H). There was no significant difference in the expression of P53 or mutant P53 by overexpression of full‐length UNC5B or UNC5B truncates (Figure 3E). The expression of BCL‐2 was increased in UN‐5637, but not in UN‐T24cells.

Through the mass spectral analysis, the ribosomal proteins were identified as the most important protein group interacting with UNC5B to promote proliferation, and BCL‐2 was identified as a target protein for regulating apoptosis. In view of the characteristics of the UNC5B truncates (containing caspase‐3 site and death domain), we also examined the binding of UNC5B to caspase‐3 and cleaved caspase‐3. No significant difference in the expression of caspase‐3 or cleaved caspase‐3 was found by CO‐IP in both groups (Figure 3F). On the contrary, more binding of S6 ribosomal protein was identified to interact with UNC5B truncates and full‐length UNC5B. Through the normalization of whole‐cell CO‐IP lysates (Figure 3G), we found that the overexpressed UNC5B and its truncates had stronger binding to S6 ribosomal proteins, especially in 399′5637, 412′5637, 399′T24 and 412′T24 cells. The proliferation‐promoting effect of ribosomal protein was also confirmed by mice xenograft models (Figure 3H,I). We observed that the overexpression of UNC5B truncates in 5637 cells significantly increased the tumour volume on mice. Comparatively, the tumour volume of 412′5637 cells was even larger than that of 399′5637 cells, which was consistent with the previous result in EDU assay and PPI analysis. By contrast, full‐length UNC5B slightly decreased the tumour volume by largely mediating cell cycle arrest, which was identical to our previous study.^30^ As shown in 3F, Co‐IP indicated more binding of BCL‐2 in cells overexpressing full‐length UNC5B and full‐length intracellular domain, but not in 412′5637 and 412′T24 cells. The combination of UNC5B and BCL‐2 reflected the stress response of cells to external stimuli (such as apoptosis), which was identical to the mass spectral analysis.

### 3.4 More binding of fibronectin, beta‐catenin and vimentin was identified by overexpressing UNC5B and UNC5B truncates

**FIGURE 4:**
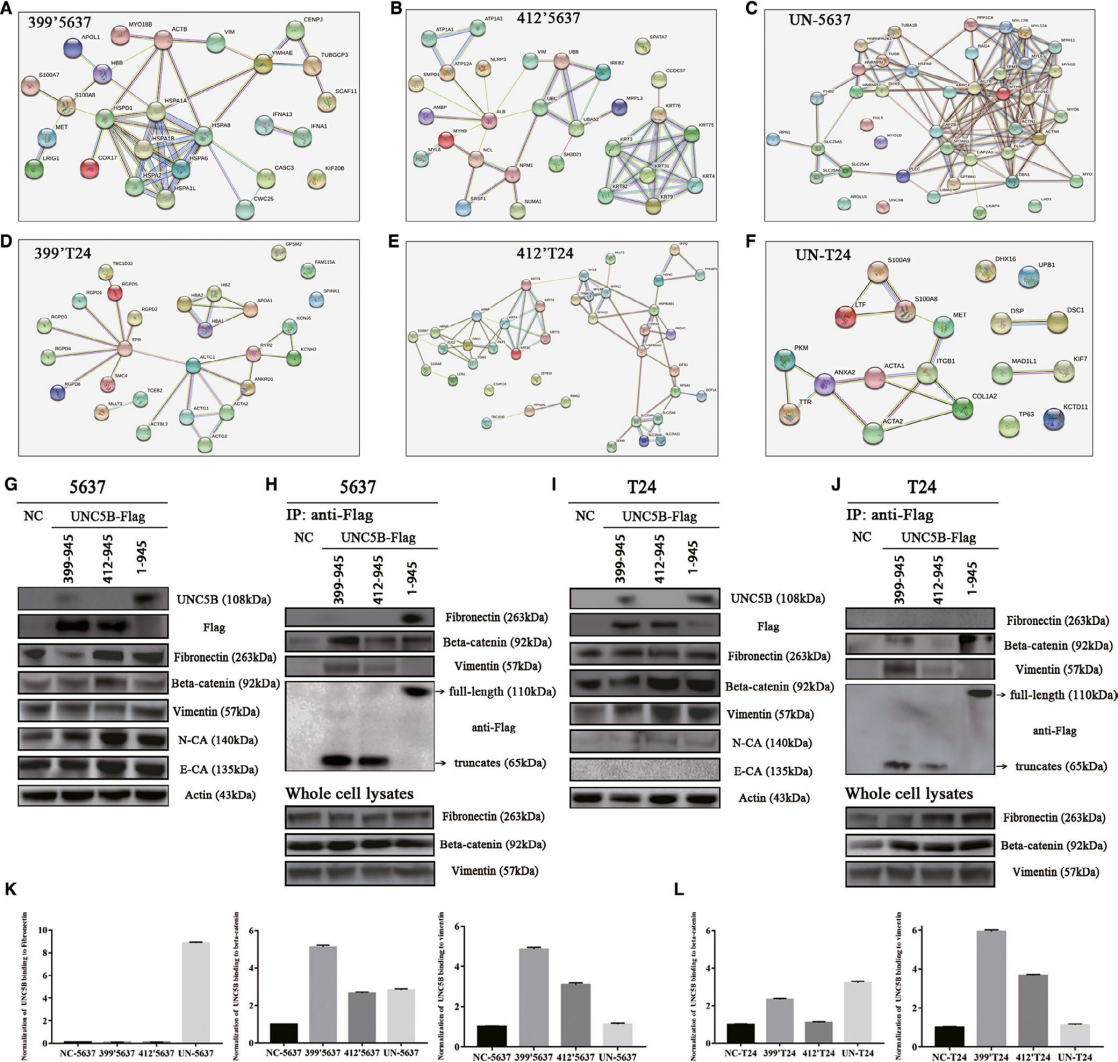
More binding of fibronectin, beta‐catenin and vimentin was identified by overexpressing UNC5B and UNC5B truncates. A‐F, Protein‐protein interaction of EMT‐related proteins was analysed by overexpressing different domains of UNC5B (A, D: residues 399‐945, B, E: residues 412‐945, C, F: full‐length UNC5B) in 5637 and T24 cells, respectively. G, WB analysis of UNC5B, fibronectin, beta‐catenin, vimentin, N‐CA and E‐CA in 5637 cells. H, Co‐IP indicated the interaction of UNC5B truncates/UNC5B with fibronectin, beta‐catenin, vimentin in 399′5637, 412′5637 and UN‐5637 cells. I, WB analysis of UNC5B, fibronectin, beta‐catenin, vimentin, N‐CA and E‐CA in T24 cells. J, Co‐IP indicated the interaction of UNC5B truncates/UNC5B with beta‐catenin and vimentin in 399′T24, 412′T24 and UN‐T24 cells. K, The normalization of UNC5B binding to fibronectin, beta‐catenin and vimentin in 5637 group of cells (left to right). L, The normalization of UNC5B binding to beta‐catenin and vimentin in T24 group of cells (left to right)

Through the mass spectrometry, a variety of epithelial‐mesenchymal transition (EMT) related proteins were co‐immunoprecipitated and identified, including fibronectin, beta‐catenin, vimentin, myosin, plectin, capping protein, hornerin, desmoplakin and. PPI analysis was applied to reveal the interaction of these differentially expressed proteins among groups (Figure 4A‐F). According to the relative protein expression of mass spectrometry, fibronectin, beta‐catenin and vimentin were examined to reveal the regulatory mechanism. WB analysis indicated that the expression of fibronectin and beta‐catenin was increased in 412′5637 cells (Figure 4G). The expression of fibronectin was also up‐regulated in UN‐5637 cells. The transfection of UNC5B truncates and full‐length UNC5B increased overall expression of N‐CA and E‐CA in 5637 cells. The binding of fibronectin, beta‐catenin and vimentin to UNC5B truncates and full‐length UNC5B were also found to be up‐regulated by CO‐IP test (Figure 4H).

The transfection of UNC5B truncates and full‐length UNC5B similarly increased the expression of fibronectin, beta‐catenin and vimentin (Figure 4I) in T24 cells. The overall expression of N‐CA was also slightly up‐regulated in T24 transfected cells. Similarly, more binding of vimentin and beta‐catenin were also co‐immunoprecipitated in 399’T24 and UN‐T24 cells (Figure 4J). Through the normalization of whole‐cell CO‐IP lysates (Figure 4K,L), more binding of fibronectin, beta‐catenin and vimentin to UNC5B and UNC5B truncates was examined.

### 3.5 Overexpression of UNC5B and UNC5B truncates promoted cell migration in vitro and tumour metastasis in vivo

**FIGURE 5:**
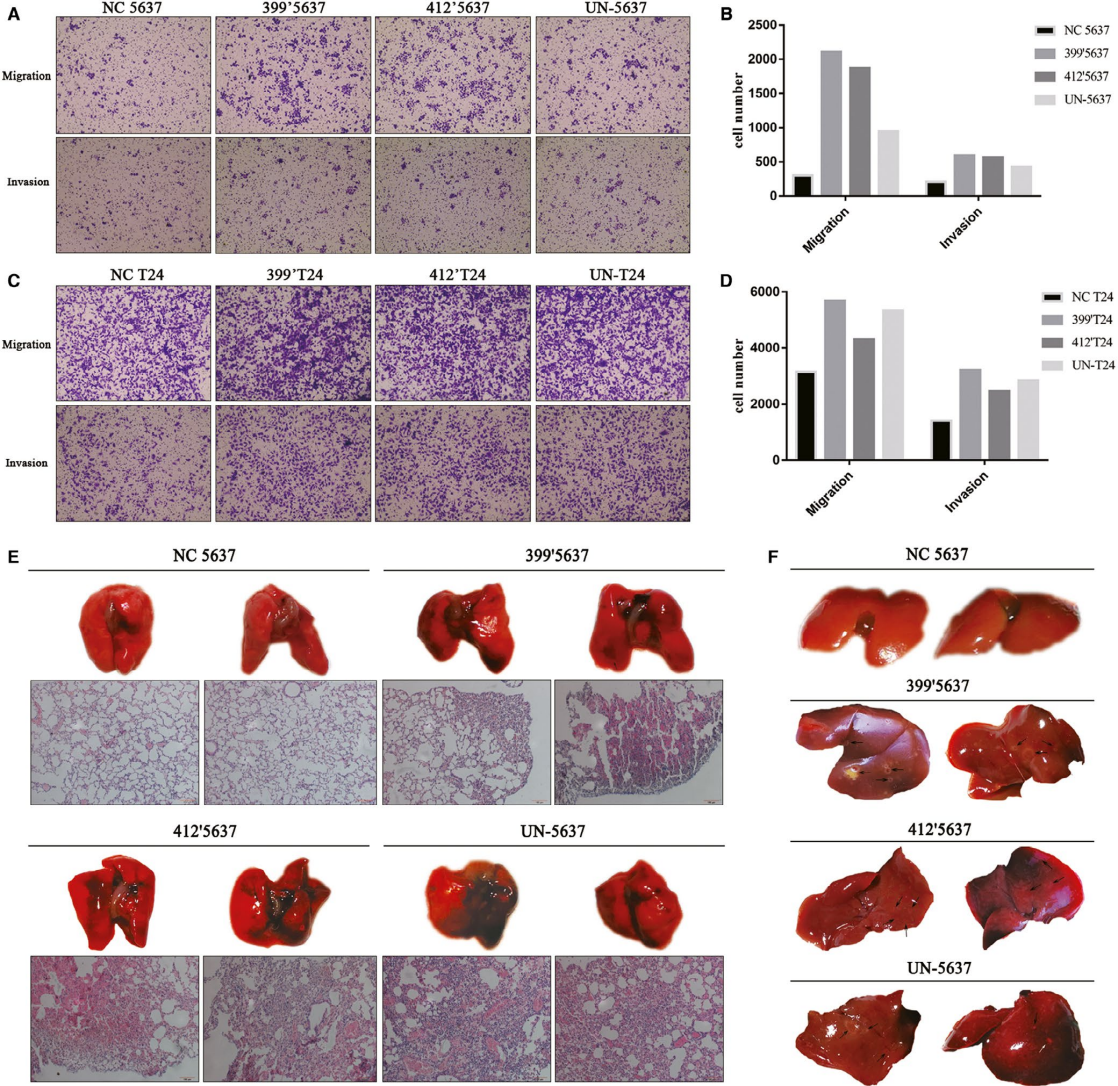
Overexpression of UNC5B and UNC5B truncates promoted cell migration tumour metastasis. A, Cell migration and invasion in NC‐5637, 399′5637, 412′5637 and UN‐5637 cells. B, Column statistical chart of the cell number on migration and invasion in 5637 group of cells. C, Cell migration and invasion in NC‐T24, 399′T24, 412′T24 and UN‐T24 cells. D, Column statistical chart of the cell number on migration and invasion in T24 group of cells. E, Pulmonary metastases and lung histology of HE staining were seen in mice after the tail vein injection of 399′5637, 412′5637 and UN‐5637 cells, respectively. Negative image of pulmonary metastases and HE staining on lung histology was only seen in mice injected of NC‐5637 cells (Top left). F, Typical image of hepatic metastases on the mice by the tail vein injection of related cells. Black arrows showed metastases in the livers

The increased expression of fibronectin, beta‐catenin and vimentin, along with their interaction with UNC5B promoted cell migration and invasion in both groups of cells (Figure 5A‐D). Comparatively, the invasion and migration of UN‐5637 cells were lower than that of 399′5637 and 412′5637 cells. In order to clarify the effect of UNC5B truncates and full‐length UNC5B on tumour metastasis, we also established animal models of tail vein injection in 16 mice. Eight weeks after the injection, tail vein injection of 399′5637, 412′5637 and UN‐5637 cells resulted in extensive pulmonary and hepatic metastasis in mice (Figure 5E,F). Some surface pulmonary metastases were visible and confirmed by HE staining (Figure 5E, below). By contrast, lung metastasis occurred in one of the four mice injected with NC‐5637 cells. This situation was also seen in liver metastasis in mice, as no visible metastatic nodules were found in mice injected with NC‐5637 cells (Figure 5F, top). However, obvious liver metastases were found in mice injected with other cells, especially 399’5637 cells.

## 4 DISCUSSION

As one of the receptors of Netrin‐1 family,^31^, ^32^, ^33^ UNC5B binds to Netrin‐1 through two immunoglobulin‐like domains of extracellular portion of UNC5B.^22^, ^34^ The dependence receptor theory suggests that UNC5B triggers apoptosis in the absence of Netrin‐1 or with the overexpression of UNC5B, which is mainly applicable in neuronal cells.^31^, ^32^, ^33^, ^35^ On the contrary, in the presence of Netrin‐1, UNC5B is regulated to promote the proliferation and migration in rectal cancer.^36^, ^37^

The mechanism of apoptosis mediated by UNC5B is considered in two ways: one is to interact with Death‐Associated Protein Kinase (DAPK) through death domain of UNC5B^25^, ^26^; the other is to activate caspase‐3 by participating in p53 apoptosis pathway.^18^, ^28^, ^29^ However, in our previous study, we found that the cotransfection of UNC5B and DAPK in BC cells could not bind to each other. The non‐binding of UNC5B and DAPK was attributed to the tight closed structure between the intracellular domain of ZU5‐UPA‐DD, which was supported by Wang et al.^38^

In our recent work, we found that full‐length UNC5B bound to cleaved caspase‐3 to significantly induce apoptosis in 5637 cells transfected with DAPK (Figure S2A). As the caspase‐3 site was not in the ZU5‐UPA‐DD supermodule structurally (Figure 1H), we had expected that apoptosis could be greatly mediated by the overexpression of UNC5B truncate (residue 412‐945) by activating cleaved capase‐3. However, we found that the overexpression of UNC5B truncates did not mediate apoptosis; on the contrary, it promoted proliferation and tumour formation by binding to a large number of ribosomal proteins. Remarkably, 412′5637 and 412′T24 cells had even stronger proliferation than 399′5637 and 399′T24 cells both in vitro and in vivo, suggesting that the caspase‐3 cleavage site (site 412) was not activated. In accordance with this, more ribosomal proteins were identified by the overexpression of UNC5B residue 412‐945 than that of UNC5B residue 399‐945 through the map analysis of mass spectrometry (Figure S1G,H). In terms of mechanism, the overexpressed UNC5B and UNC5B truncates did not bind to cleaved caspase‐3, nor did they mediated apoptosis by changing the phenotype of p53. By contrast, they increased both expression and binding of S6 ribosomal protein, thereby facilitating tumour formation in mice. These results suggested that the closed conformation of intracellular portion might blocked the cleavage site (412 site) in BC cells, which was functionally consistent with the study by Wang et al.^38^

Therefore, the effect of full‐length UNC5B and its truncates on cell proliferation was mainly mediated by their binding proteins. For full‐length UNC5B, our previous studies have confirmed that cell cycle regulation played an important role in inhibiting tumour proliferation,^19^, ^39^ among which we verified CDC14A and P53 as target proteins mediating cell cycle arrest by combining with UNC5B.^30^ However, two aspects needed to be clarified. First, due to the difference in the expression of UNC5B in BC cells, the effect of cell cycle arrest mediated by CDC14A and P53 differed. Second, in addition to CDC14A/P53 factor, a variety of UNC5B binding proteins were also found to play a competitive and antagonistic role by bio‐informatics analysis. For instance, the number of ‘negative regulation of biological process’ was 167, while the number of ‘positive regulation of biological process’ was 147. Identically, the number of ‘negative and positive regulation of cellular process’ was 135 and 118, respectively. This resulted in the relatively mild and minor anti‐cancer effect of UNC5B, which was very different from the pro‐apoptotic effect of caspase‐3 activation by UNC5B structural mutants in the study of Wang et al.^38^ Comparatively, the number and variety of proteins bound to the UNC5B truncates were very limited, among which ribosomal protein was the most proliferation‐related protein, and was proved to promote proliferation and tumour formation in this study.

Similarly using flag‐labelled lentivirus, we transfected UNC5B to Renal carcinoma cells (786‐0, ACHN), prostate cancer cells (22RV‐1) and BC cells (UMUC3) (Figure S2B), and examined the apoptosis. We found that only the BC cells (UMUC3) transfected with UNC5B had the effect of promoting apoptosis (the late apoptosis rate was increased by 5.6%) (Figure S2C). Through previous mass spectrometry and bio‐informatics analysis, no direct interaction of Netrin‐1 and UNC5B was found in BC cells. However, we found that the pro‐apoptotic effect of UNC5B was reversed by transfecting Netrin‐1 to UN‐5637 and UN‐T24 cells (Figure S2D‐F). These experiments seemed to suggest an inhibitory effect of UNC5B specifically on BC cells. Besides, although Netrin‐1 was not pulled down by CO‐IP tests, its regulatory effect on UNC5B in apoptosis was still existed, which was supported by the dependence receptor theory.

On the other hand, a large number of epithelial‐mesenchymal transition (EMT) related proteins bound to UNC5B truncates or full‐length UNC5B played a crucial role in cell movement, which included spectrin, fibronectin, beta‐catenin, vimentin, capping protein, desmoplakin and plectin. These proteins were considered to be polymerized into molecular networks and facilitate cell metastasis in various cancers.^40^, ^41^, ^42^, ^43^ Fibronectin, beta‐catenin and vimentin were chosen to verify the promoting effect of UNC5B truncates and UNC5B on migration, invasion and metastasis in this study. In the previous study, we have noted the tendency of full‐length UNC5B to promote metastasis. Here, we reported that the binding of UNC5B intracellular domain (residue 399‐945) to vimentin and beta‐catenin was even more than that of full‐length UNC5B, which was consistent with the result in migration, invasion and metastasis. Besides, spectrin family was the most detected and identified in cells transfected with UNC5B truncates. In view of the reports of spectrin in impacting tumour development and aggressiveness,^44^, ^45^, ^46^, ^47^ the binding of spectrin family to the intracellular domain of UNC5B was also considered to be closely related to the metastasis of BC cells.

From this aspect, it seems that we can speculate the mechanism of UNC5B on BC cells. We believe that UNC5B, as a membrane protein, is very low expressed in BC cells; thus, its biological effect is mainly regulated by its binding proteins. Through mass spectrometry, we found that the most binding proteins by overexpressing UNC5B and its truncates are EMT‐related proteins, with the effect of promoting cell invasion and metastasis. On the other hand, although full‐length UNC5B binds to more protein groups (such as ribosomal proteins), the overall effect on proliferation is inhibited. Through our previous study, we confirmed that full‐length UNC5B combined with a large number of inhibitors to mediate cell cycle arrest and apoptosis, in which its binding to p53 and CDC14A played an important role.^30^ However, the binding ability of UNC5B truncates is much lower than that of full‐length UNC5B, which hardly binds to protein groups except for ribosome and histone, thus promoting cell proliferation. To sum up, the biological effect of UNC5B on BC is determined by its binding proteins. Because of the diversity of UNC5B binding protein and its potential for metastasis of BC cells, it is not considered as a typical tumour suppressor gene in BC.

In this study, we reveal that overexpressing the intracellular domains of UNC5B cannot bind or activate cleaved caspase‐3 to trigger apoptosis. On the contrary, the intracellular domains of UNC5B promote cell proliferation in vitro and tumour formation in vivo, by binding a large number of ribosomal proteins. They also facilitate BC cells to migrate, invade and metastasize by interacting with EMT‐related proteins, such as fibronectin, beta‐catenin and vimentin.

## CONFLICT OF INTEREST

The authors declare no conflicts of interest.

## AUTHOR CONTRIBUTIONS

**Yexiang Huang:** Conceptualization (lead); Data curation (lead); Formal analysis (lead); Funding acquisition (equal); Investigation (lead); Methodology (lead); Project administration (equal); Resources (equal); Software (lead); Supervision (equal); Validation (lead); Visualization (equal); Writing‐original draft (lead); Writing‐review & editing (lead). **Zhe Zhang:** Conceptualization (equal); Investigation (equal); Methodology (supporting); Project administration (equal); Resources (supporting); Supervision (lead); Validation (equal); Visualization (supporting). **Miao Miao:** Software (equal). **Chuize Kong:** Conceptualization (equal); Funding acquisition (lead); Investigation (lead); Project administration (lead); Resources (lead); Supervision (lead).

## Supporting information

Fig S1

Fig S2

## Data Availability

The data sets used and analysed in this study are available from the corresponding author upon reasonable request.
