# Supplementary figures and images for: The intracellular domain of UNC5B facilities proliferation and metastasis of bladder cancer cells
Source: J Cell Mol Med. 2020 Dec 20;25(4):2121–35. doi: 10.1111/jcmm.16172 (PMC7882925; doi:10.1111/jcmm.16172)

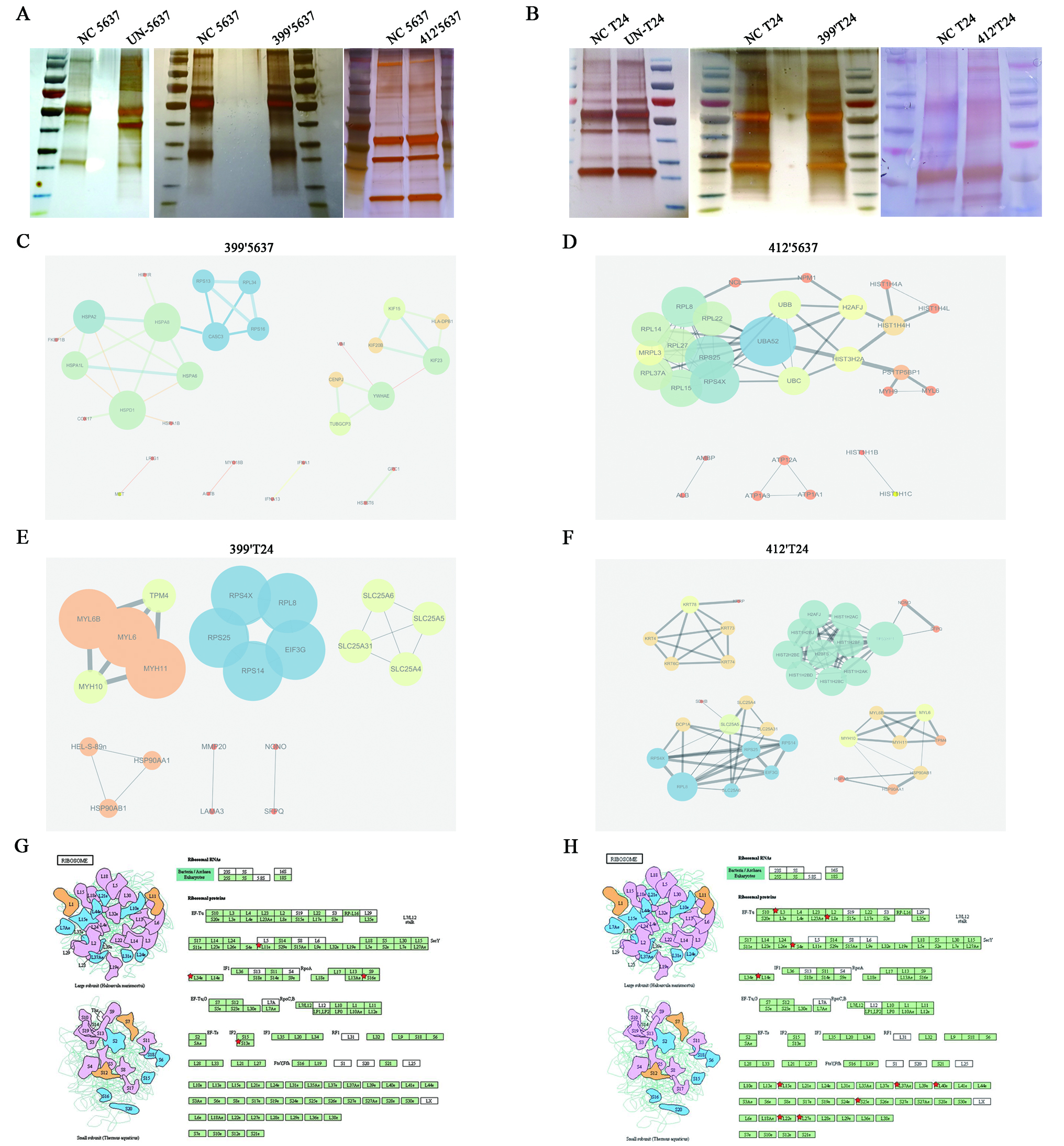

Supplement: Supplementary file 1 — Fig S1 [file JCMM-25-2121-s001.jpg]

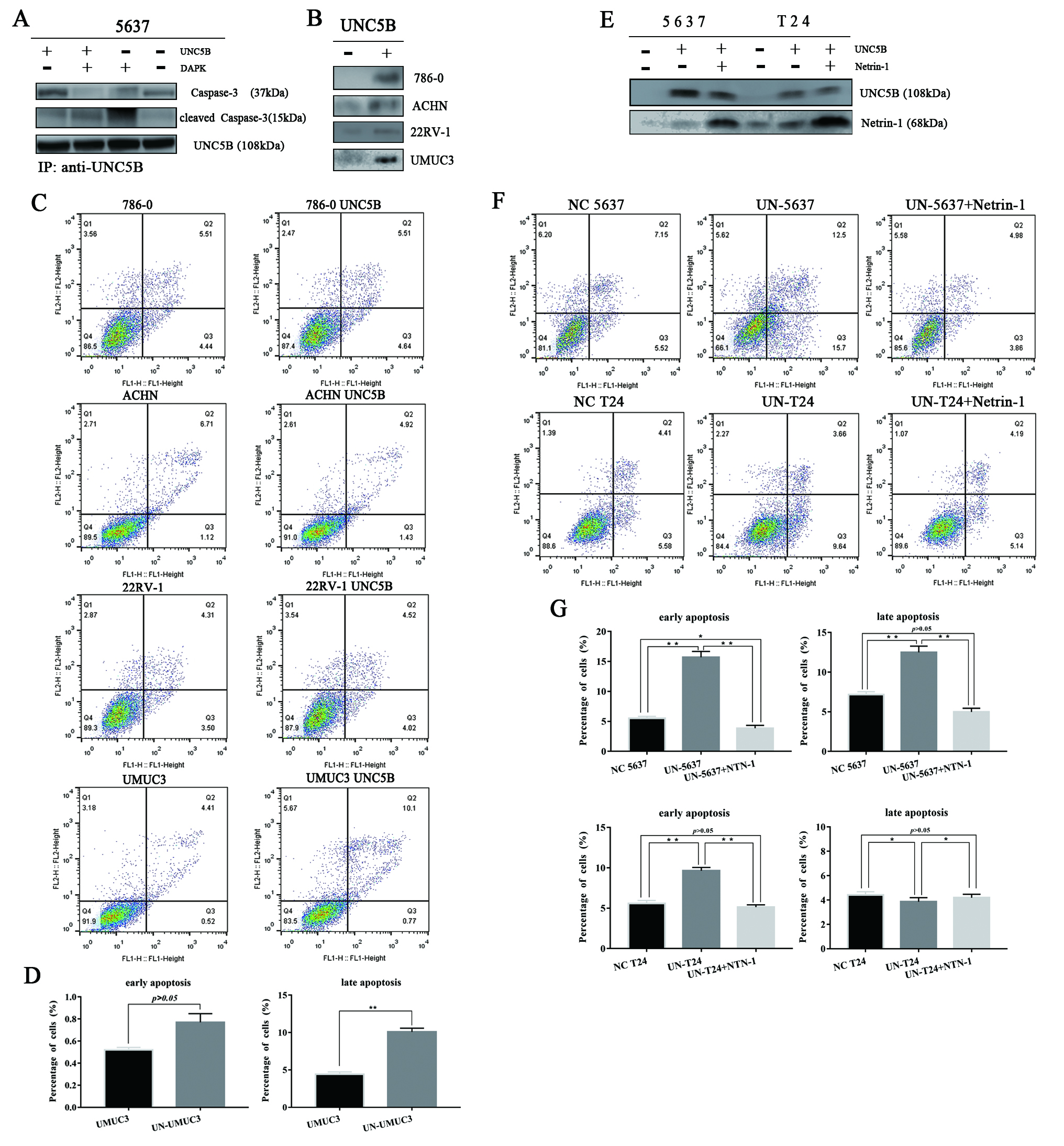

Supplement: Supplementary file 2 — Fig S2 [file JCMM-25-2121-s002.jpg]
